# MicroRNA expression profile analysis in sperm reveals hsa-mir-191 as an auspicious omen of in vitro fertilization

**DOI:** 10.1186/s12864-020-6570-8

**Published:** 2020-02-17

**Authors:** Hua Xu, Xin Wang, Zhikai Wang, Jianhui Li, Zhiming Xu, Maohua Miao, Guowu Chen, Xiangdong Lei, Jun Wu, Huijuan Shi, Ke Wang, Tiancheng Zhang, Xiaoxi Sun

**Affiliations:** 1 Shanghai JiAi Genetics & IVF Institute, Obstetrics and Gynecology Hospital, Fudan University, No.588 Fangxie Road, Shanghai, 200011 China; 2 NHC Key Lab. of Reproduction Regulation(Shanghai Institute of Planned Parenthood Research), Hospital of SIPPR, Fudan University, Shanghai, China; 3 NHC Key Lab. of Reproduction Regulation(Shanghai Institute of Planned Parenthood Research), Public School, Fudan University, Shanghai, China; 4 Key Laboratory of Female Reproductive Endocrine Related Diseases, Obstetrics and Gynecology Hospital, Fudan University, Shanghai, 200011 China; 5 Department of Obstetrics and Gynecology, Zhongshan Hospital, Fudan University, Shanghai, 200032 China; 6 NHC Key Lab. of Reproduction Regulation(Shanghai Institute of Planned Parenthood Research), Pharmacy School, Fudan University, No.2140 xietu road, xuhui district, Shanghai, People’s Republic of China

**Keywords:** miRNA, Hsa-mir-191, Hsa-mir-34c, RNA-Seq, Embryonic development

## Abstract

**Background:**

MicroRNAs (miRNAs) are a class of noncoding small RNAs that play important roles in many physiological processes by regulating gene expression. Previous studies have shown that the expression levels of total miRNAs increase during mouse embryonic development, and some miRNAs control the regulatory network in development progression. However, few studies have focused on the effects of miRNAs on early human embryonic development. The relationship between miRNAs and early human embryogenesis is still unknown.

**Results:**

In this study, RNA-seq data collected from sperm samples from 102 patients with a normal sperm index but treated with assisted reproductive technology (ART) were analyzed for the relationships between differentially expressed small RNAs and the fertilization rate (FR), blastocyst rate and high-quality embryo rate (HQER). The sperm samples with high hsa-mir-191 expression had a higher FR, effective embryo rate (EER) and HQER. hsa-mir-191 was used as a single indicator to predict the HQER. The receiver operating characteristic (ROC) curve had an area under the ROC curve (AUC) of 0.686. We also found that hsa-mir-191 expression is correlated with an abnormal sperm rate (cor = 0.29, *p* < 0.01). We also evaluated the relationship between hsa-mir-34c and early human embryo development in these 102 sperm samples and obtained negative results.

**Conclusions:**

These findings suggest that high hsa-mir-191-5p expression in sperm is associated with early human embryonic quality and that hsa-mir-191-5p could be used as a potential marker to screen high-quality sperm to improve the success rates of in vitro fertilization (IVF).

## Background

MicroRNAs (miRNAs) are a class of noncoding small RNAs 19–24 nt in length, which, after transcription, bind to the 3′-untranslated region of the target mRNA, causing downregulation of the target gene. Previous studies have shown that the expression characteristics of miRNAs are closely related to cell morphology, disease progression, cell differentiation, and gamete maturation [1–3]. However, there are few studies on the relationship between miRNAs and early human embryo development.

The scientific view of the role of the sperm content in the reproductive process is constantly evolving. Early studies suggested that sperm played a role only in transmitting the paternal genome during the reproductive process. However, an increasing number of studies have shown that the role of sperm in the reproductive process is diversified. The sperm content also contains many coding and noncoding RNAs [4] that play an important role in epigenetics [5, 6].

Epigenetics plays an important role in the early development of embryos, especially miRNAs carried by sperm. Previous studies have also shown that miRNAs in mouse sperm respond to benzo-a-pyrene exposure and reflect gene expression in early developing mouse embryos [7, 8]. Liu et al. observed that mir-34c inhibitors blocked pronuclear fusion in most embryos, but Jingwen Wu et al. did not observe pronuclear fusion defects in single mir-34c knockout mice [9, 10]. Pri-miR-181c is carried into the oocyte, and mature miR-181 plays an important role in stem cell and embryo development [11]. The miR-34 family in bovine sperm is also required for the development of bovine female gametes or male gametes [12]. Increasing the expression of miR-34c in somatic cell nuclear transfer (SCNT) embryos does not only affect the early development of bovine embryos and increase the cleavage rate of developing embryos but also changes the quality of the resulting SCNT embryos [13]. However, all these studies used animal models, such as mice or cattle, to assess the role of miRNAs in early embryonic development. In a previous study, our team collected semen samples from 87 patients with IVF and demonstrated their piRNA, tsRNA, rsRNA and miRNA expression profiles [14].

In this work, using 102 human sperm samples containing an additional 15 samples, we focused on differential expression analysis of miRNA. Our results reveal that expression of a series of miRNAs are correlated with sperm quality and embryo quality before implantation. Meanwhile, we also evaluated the role of the controversial miR-34 family, which comprises the most previously reported sperm-carrying miRNAs that play an important role in the early embryonic development of mice/bovines following in vitro fertilization (IVF) in humans. These results will help us further understand the function of sperm-borne miRNAs in embryo development.

## Results

### Overview of small RNA library sequencing in spermatozoa and sample grouping

**Fig. 1:**
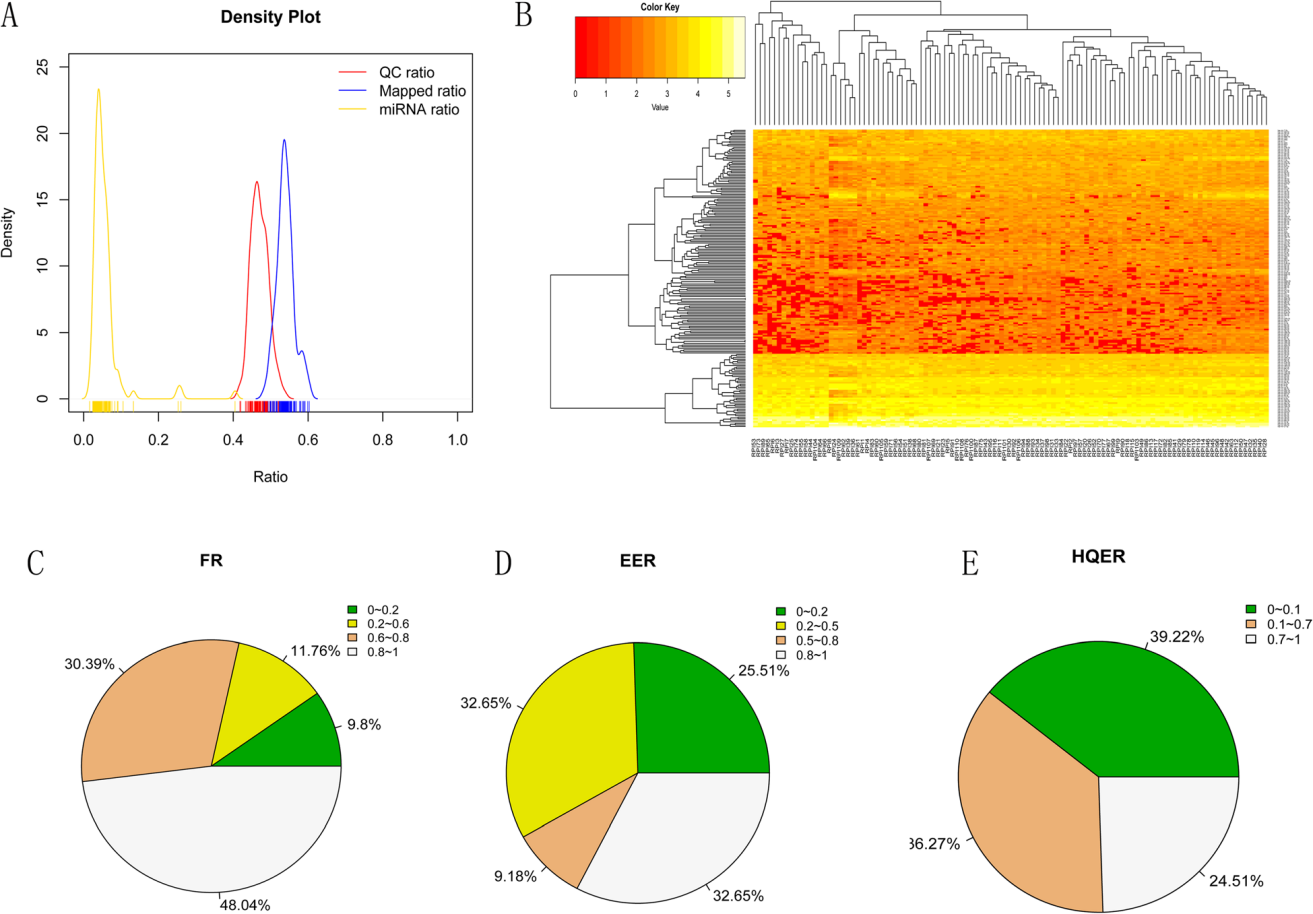
miRNA profile of IVF patient sperm: **a** Sequencing data quality control (red line), mapping (blue line) and miRNA annotation (yellow line). The ordinate indicates the number of samples, and the abscissa indicates the ratio of the reads obtained by the corresponding analysis to the total reads before the analysis. **b** Heat map of miRNA expression characteristics. Yellow represents low expression, and red represents high expression. The abscissa in the heat map shows the sample name, and the ordinate shows the miRNA name. c-e Patients were grouped according to their FR, EER and HQER

To evaluate the role of miRNAs in spermatozoa in the process of embryo development during IVF, miRNA profiles in the spermatozoa of 102 patients who underwent IVF were investigated by small RNA deep sequencing. Quality control (QC) assessment showed that approximately 50% of reads were filtered after QC. Fifty percent of reads after QC could be mapped to the human reference genome (hg19). However, most of the samples had only 5% mappable reads that could be annotated for known miRNAs (Fig. 1a). We identified a total of 797 of 2042 known human miRNAs. Hierarchical cluster analysis indicated that the miRNA expression levels were significantly different in 102 samples (Fig. 1b). Samples were divided into different groups based on the FR(fertilization rate), EER(effective embryo rate) and HQER(high-quality embryo rate) to detect differentially expressed microRNAs. Based on the FR, we divided the sequencing results into four groups: patients with an FR < 20%, accounting for 9.8% of all patients; patients with an FR between 20 and 60%, accounting for 11.76% of all patients; patients with an FR between 60 and 80%, accounting for 30.39% of all patients; and patients with an FR > 80%, accounting for 48.04% of all patients (Fig. 1c). Based on the EER, we divided the sequencing results into four groups: patients with an EER < 20%, accounting for 25.51% of all patients; patients with an EER between 20 and 50%, accounting for 32.65% of all patients; patients with an EER between 50 and 80%, accounting for 9.18% of all patients; and patients with an EER > 80%, accounting for 32.65% of all patients (Fig. 1d). Based on the HQER, we divided the sequencing results into three groups: patients with an HQER < 10%, accounting for 39.22% of all patients; patients with an HQER between 10 and 70%, accounting for 24.51% of all patients; and patients with an HQER > 70%, accounting for 36.27% of all patients (Fig. 1e). We demonstrated that most patients have a high FR but not enough embryos. This result may indicate that there is no direct link between fertility and embryo development.

### Analysis of differentially expressed miRNAs

**Fig. 2:**
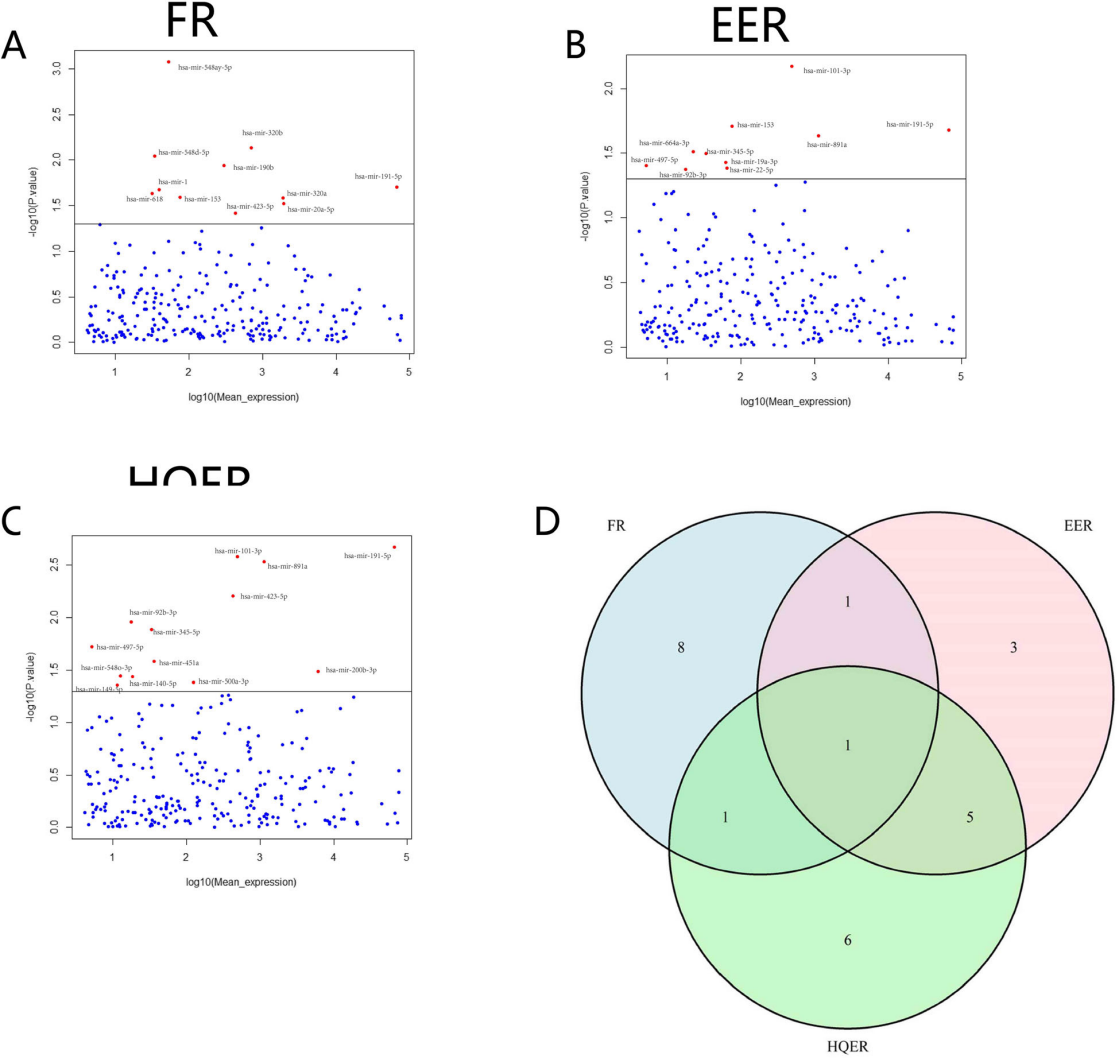
Differential expression analysis of miRNAs in sperm: (**a-c)** Differentially expressed miRNAs were screened in different FR, EER and HQER subgroups, with red dots indicating differentially expressed miRNAs and blue dots indicating nondifferentially expressed miRNAs. **d** Differential expression of hsa-mir-191 in the FR, EER and HQER groups (IVF embryo development). The *p* value corresponding to the baseline in the figure is 0.05, and the red dots above the baseline corresponds to the different points

A total of 11 miRNAs were identified as differentially expressed, with intergroup differences in the FR (*p* < 0.05): hsa-mir-191-5p, hsa-mir-320a, hsa-mir-320b, hsa-mir-190b, hsa-mir-423-5p, hsa-mir-20a-5p, hsa-mir-548ay-5p, hsa-mir-153, hsa-mir-548d-5p, hsa-mir-1 and hsa-mir-618 (Fig. 2a). A total of 10 miRNAs were identified as differentially expressed, with intergroup differences in the EER (*p* < 0.05): hsa-mir-191-5p, hsa-mir-891a, hsa-mir-101-3p, hsa-mir-345-5p, hsa-mir-664a-3p, hsa-mir-19a-3p, hsa-mir-92b-3p, hsa-mir-153, hsa-mir-22-5p and hsa-mir-497-5p (Fig. 2b). A total of 13 miRNAs were identified as differentially expressed, with intergroup differences in the HQER (*p* < 0.05): hsa-mir-191-5p, hsa-mir-200b-3p, hsa-mir-891a, hsa-mir-500a-3p, hsa-mir-423-5p, hsa-mir-101-3p, hsa-mir-345-5p, hsa-mir-92b-3p, hsa-mir-140-5p, hsa-mir-548o-3p, hsa-mir-149-5p, hsa-mir-451a and hsa-mir-497-5p (Fig. 2c and Supplementary Table S1). By overlapping three sets of differentially expressed miRNAs, we identified only one miRNA, hsa-mir-191-5p, that was differentially expressed in the FR, EER and HQER groups (Fig. 2d). This result indicates that hsa-mir-191-5p may be a key factor in both fertility and embryo development.

### Comparison of miR-191-5p expression in different FR, EER and HQER groups

**Fig. 3:**
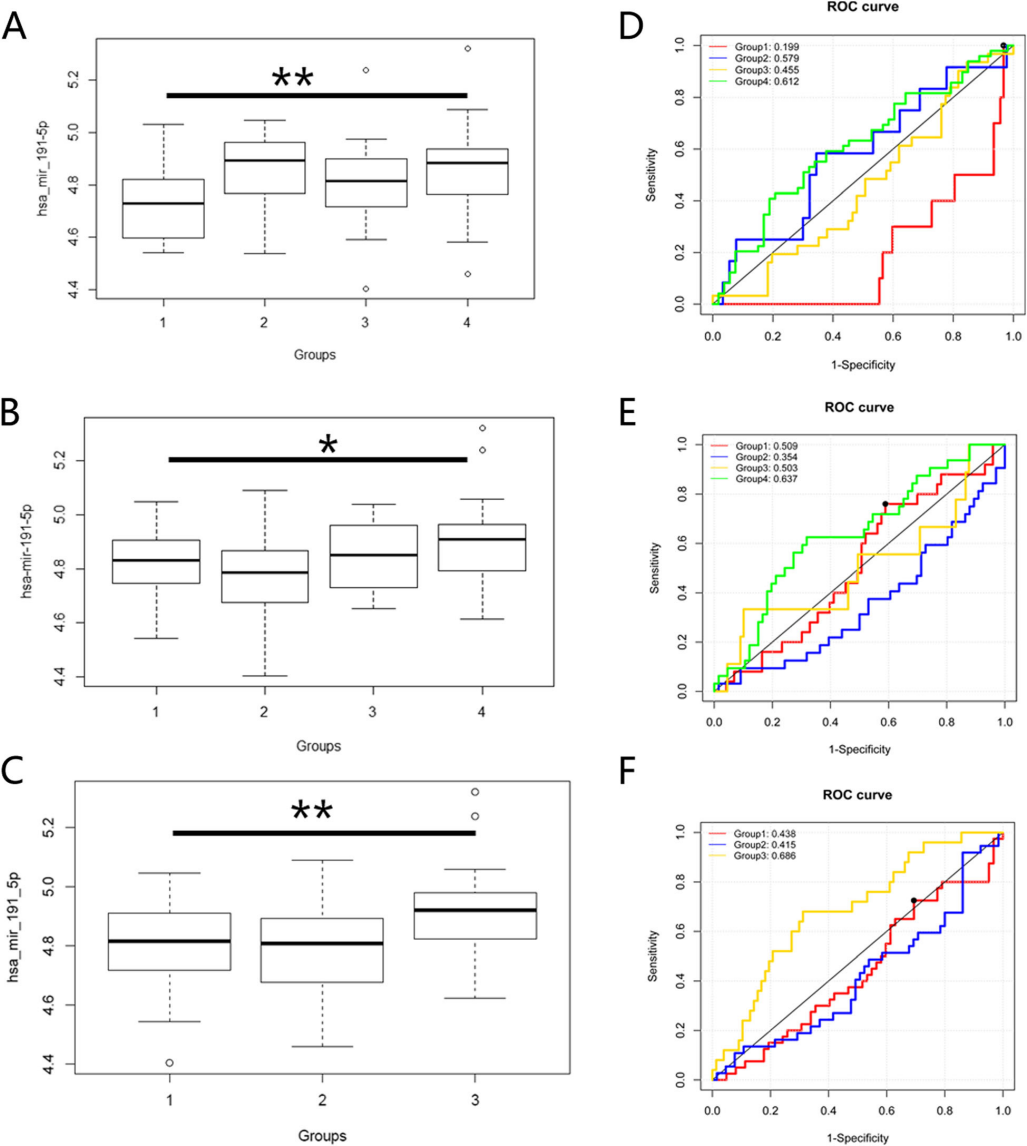
Comparison of hsa-mir-191-5p expression: **a** Differential expression of hsa-mir-191-5p in sperm samples from patients with different FRs. **b** Differential expression of hsa-mir-191-5p in sperm samples from patients with different EERs. **c** Differential expression of hsa-mir-191-5p in sperm samples from patients with different HQER. **d** When hsa-mir-191-5p was used as a single predictor, the ROC curves of patients in different FR groups were obtained. **e** When hsa-mir-191-5p was used as a single predictor, the ROC curves of patients in different EER groups were obtained. **f** When hsa-mir-191-5p was used as a single predictor, the ROC curves of patients in different HQER groups were obtained

We used the independent T test to compare differences in hsa-mir-191-5p expression in the FR, EER and HQER groups. The results showed that the expression of hsa-mir-191-5p in the highest FR group was significantly higher than that in the lowest FR group (*p* < 0.01) (Fig. 3a). The expression of hsa-mir-191-5p was significantly higher in the highest EER group (*p* < 0.05) (Fig. 3b), but there was no significant difference between the other EER groups. In the highest HQER group, the expression of miR-191-5p was significantly higher than that of the lowest HQER group (*p* < 0.01) (Fig. 3c), but there was no significant difference between the other HQER groups. This finding suggests that hsa-mir-191-5p expression in patients with a high FR, EER and HQER was higher than that in those with a low FR, EER and HQER following IVF. To further clarify the function of hsa-mir-191-5p during fertilization and embryonic development, we conducted receiver operating characteristic (ROC) curve analysis. The results showed that the area under the ROC curve (AUC) for the FR, EER and HQER groups predicted by hsa-mir-191-5p was 0.612, 0.637 and 0.686, respectively, which indicates that miR-191-5p could be used to predict whether the patients belonged to the highest FR, EER and HQER groups, especially in the high HQER group, with an AUC close to 0.7 (Fig. 3d, e, f). In addition, the results further indicated that high hsa-mir-191-5p expression could lead to improved embryo quality, although its low expression did not indicate that the IVF results would be poor.

### Analysis of correlations between hsa-mir-191-5p and routine sperm parameters

**Fig. 4:**
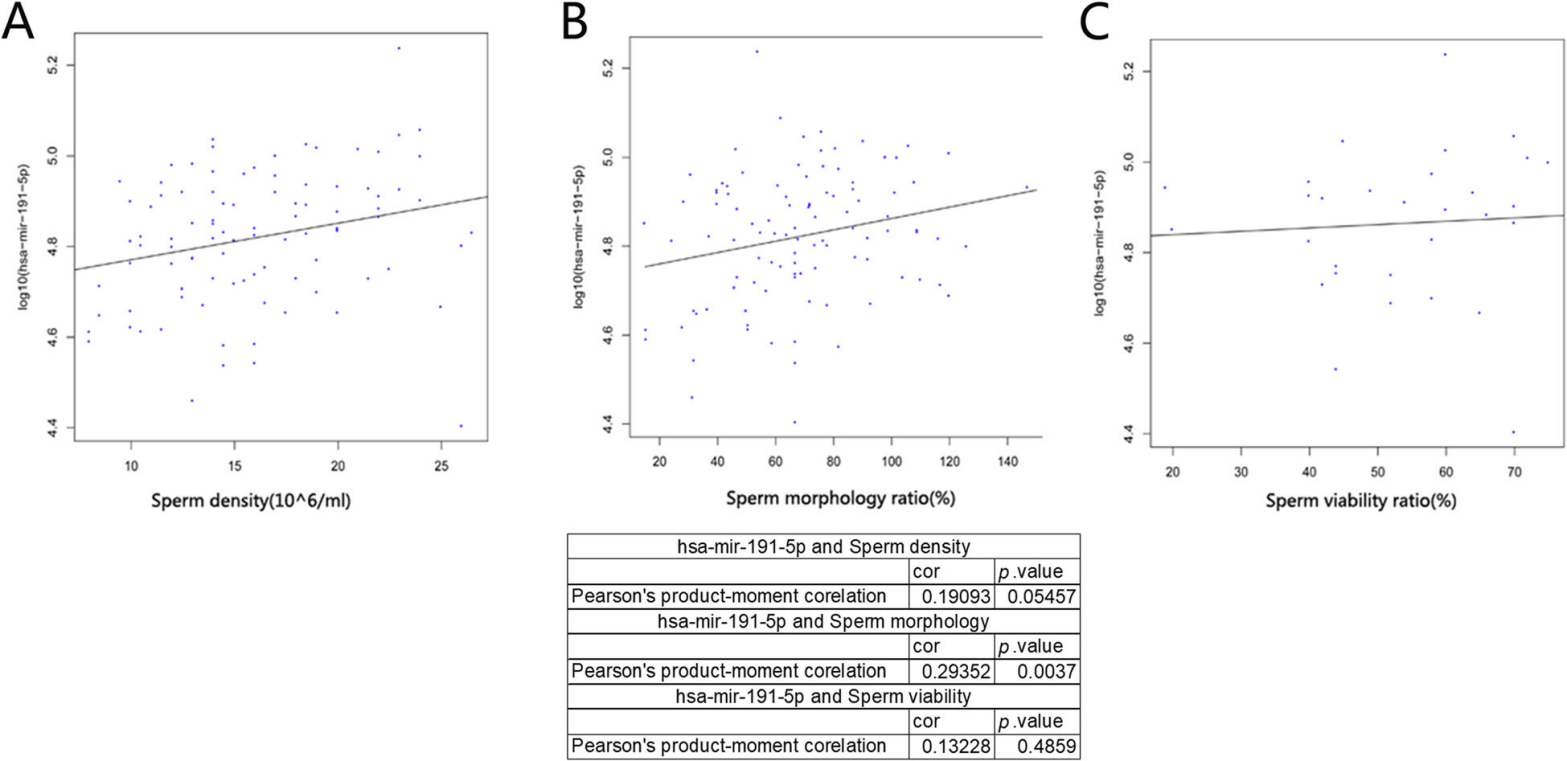
Analysis of the relationship between the expression of hsa-mir-191-5p and routine sperm parameters: **a** The relationship between the expression level of hsa-mir-191-5p and sperm density was calculated by the Pearson method. **b** The relationship between the expression level of hsa-mir-191-5p and sperm morphology was calculated by the Pearson method. **c** The relationship between the expression level of hsa-mir-191-5p and sperm viability was calculated by the Pearson method

We investigated the correlation between hsa-mir-191-5p and 3 sperm routine parameters: sperm density, sperm morphology and sperm viability. The results showed that the correlation between hsa-mir-191-5p and sperm density as well as sperm viability was not significant (Fig. 4a and c, *p* > 0.05), while the correlation between hsa-mir-191-5p and sperm morphology was significant (Fig. 4b, *p* < 0.01), but the correlation coefficient was only 0.29, indicating a weak correlation between the two. As shown above, hsa-mir-191-5p may be one of the key molecules involved in maintaining normal sperm morphology.

### Overview evaluation of the function of sperm-carrying hsa-mir-34c in the early development of human embryos

**Fig. 5:**
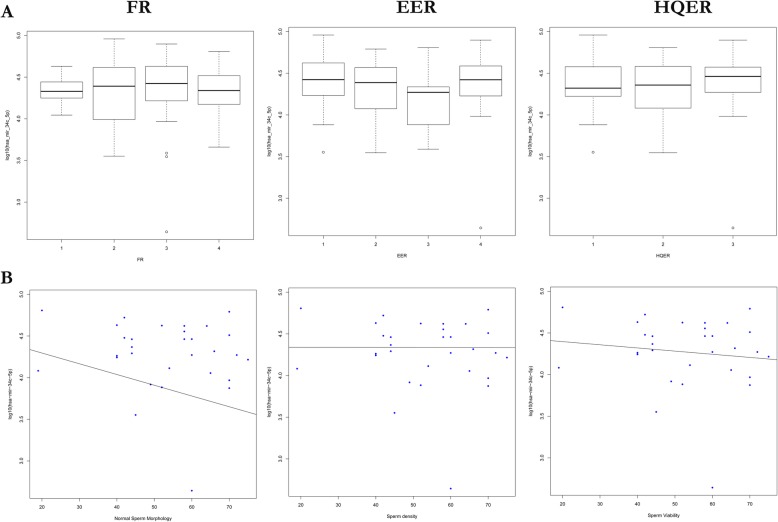
Expression characteristics of hsa-mir-34c: **a** Differential expression of hsa-mir-34c between different FR, EER and HQER groups. **b** Correlation analysis between hsa-mir-34c and sperm density, morphology and viability

The function of sperm-carrying mmu-mir-34c in early embryonic development has been discussed in mice, but its function in the early development of human embryos has not been studied. In this study, our results showed no significant difference in hsa-mir-34c between different FR, EER and HQER groups (Fig. 5a). We also investigated the correlation between hsa-mir-34c and three routine sperm parameters, namely, sperm density, sperm morphology and sperm viability (Fig. 5b). The results showed that hsa-mir-34c had a weak negative linear relationship with sperm morphology (*p* < 0.05) but was not linearly related to sperm density or sperm viability(Table 1). This evidence does not indicate that sperm-carrying hsa-mir-34c plays an important role in the early development of human embryos. However, hsa-mir-34c may have a certain function during the normal development of sperm morphology.

**Table 1:** Correlation anaylsis between sperm parameters and microRNAs expression

	sperm density		sperm morphology		sperm viability	
	cor	P.value	cor	P.value	cor	P.value
**Pearson’s product-moment correlation**	**−0.0709**	**0.4791**	**−0.2252**	**0.0265**	**−0.1717**	**0.3642**
**Spearman’s rank correlation rho**	**−0.026**	**0.7951**	**−0.2288**	**0.0242**	**−0.1611**	**0.395**
**Kendall’s rank correlation tau**	**−0.0156**	**0.817**	**−0.1556**	**0.0261**	**−0.1153**	**0.3801**

## Discussion

The process of artificial assisted reproductive technology (ART) is affected by many factors, and the clinical success rate is only approximately 30%, which brings economic, physical and mental burdens to the majority of patients and in turn has a great impact on the outcome of IVF. If high-quality embryos can be selected before embryo transfer, a reasonable interval between embryo development and implantation can be given to reduce the rejection of the uterus and improve the success rate of IVF (i.e., ART). Recent studies have shown that the sperm content plays an important role in the development of early embryos. When the sperm content is used as a biomarker, the preferential selection of high-quality sperm can effectively improve early embryonic development and further affect pregnancy outcomes. In this study, high-throughput sequencing was used to deeply sequence the miRNAs carried by a large number of IVF-treated male sperm. The results showed that sperm with high hsa-mir-191 expression had better early embryo development than sperm with low hsa-mir-191 expression, making it possible to improve the success rate of ART through miRNA-based sperm screening.

hsa-mir-191 is believed to belong to the same family as hsa-mir-425, which is located in the first intron of the DALRD3 gene on human chromosome 3 (3p21.31) and encodes four mature miRNAs: hsa-mir-191-5p, hsa-mir-191-3p, hsa-mir-425-5p and hsa-mir-425-3p [15]. Since hsa-mir-191 is located 381 bases upstream of hsa-mir-425, both miRNAs are generally transcribed simultaneously. However, current studies have shown that hsa-mir-191 expression is significantly higher than hsa-mir-425 expression in various tissues [16, 17]. It has been reported that hsa-mir-191 is abnormally expressed in several cancers and various other diseases, such as type 2 diabetes, Crohn’s disease, pulmonary hypertension and Alzheimer’s disease. However, only a few reports have shown that hsa-mir-191 is involved in the reproductive process of humans. Sharma S et al. reported that hsa-mir-191-5p targets the SOX4 gene and is a key signaling pathway in oncogenesis [18]. Moreover, Camilla M. Whittington et al. reported the downregulation of SOX4, a transcription factor, during early pregnancy in the uteri of both *Monodelphis domestica* and *Sminthopsis crassicaudata* [19]. Our results show that the level of hsa-mir-191-5p in sperm is closely related to the process of fertilization and early development of the embryo. These results suggest that hsa-mir-191-5p/SOX4 may play an important role in early embryonic development. In addition, studies by Grinchuk OV et al. have also shown the association of hsa-mir-191, hsa-mir-425, DALRD3 and NDUFAF3 with spermatogenesis. These genes have significant coexpression relationships in the sperm cells of normal individuals, whereas their direct coexpression relationship is not significant in patients with teratozoospermia [20]. Other studies have found that hsa-mir-191 has a higher concentration in the IVF/intracytoplasmic sperm injection (ICSI) cycle failure medium than in the successful medium [21]. This finding further illustrates the important role of hsa-mir-191 in embryonic development. Previous contradictory studies in mice have suggested that parental mmu-mir-34c is important for the first cleavage [9]. However, it has also been reported that the first cleavage in male mmu-mir-34c-knockout mice is normal [10]. The effect of hsa-mir-34c on early embryo development in human sperm has not been reported. In the present study, we revealed no difference in human hsa-mir-34c expression between different FR (fertilization rate), EER (effective embryo rate) and HQER (high-quality embryo rate) groups and no strong correlation between the amount of hsa-mir-34c expression and routine sperm parameters. This result suggests that hsa-mir-34c in human sperm may affect neither sperm quality nor embryo quality.

How to select high quality single sperm is an important issue that we often encounter in clinical practice. Recently, the FISH-Flow technique reported by Riccardo Arrigucci et al. makes it possible to analyze single cells by flow cytometry and RNA probes [22]. Rachel L. Harris et al.’s FISH-TAMB technology further implements labeling of mRNA in live cells [23]. If these technologies can be combined with highly sensitive miRNA-191 probe [24], it will enable to help us implement the miRNA-191 probe as a sorting label for sperm selection. Merck Millipore’s SmartFlare™ has provided some commercial methods for flow detection of live cellular RNA, which has inspired us to further apply this concept to assisted reproductive technologies and assess the safety and effectiveness of miRNA-191 probe.

## Conclusions

This study revealed differences in the miRNA expression profiles of the FR, EER, and HQER groups and suggested that hsa-mir-191-5p expression in patients with a high FR, EER and HQER who underwent IVF was higher than in those with a low FR, EER and HQER, highlighting a possible role for hsa-mir-191-5p in IVF and embryonic development and suggesting that hsa-mir-191-5p could play a key role in maintaining normal sperm morphology. These results suggest that hsa-mir-191-5p could be used as a potential biomarker to improve the success rate of IVF.

## Methods

### Sample collection and embryo evaluation

We recruited 102 couples at Shanghai Jiai Genetics and IVF Institute between May 2011 and December 2012. All couples were in the first IVF cycle. Semen samples were obtained from male participants by masturbation 3 days after sexual abstinence. The sample was added to the PureSperm System, and sperm cells were obtained after centrifugation at 500 g for 20 min at 25 °C. All semen samples were analyzed for primary semen parameters, including sperm density, motility, viability and morphology, according to the WHO Semen Analysis Manual (5th edition, 2010) to ensure that the recruited males provided normal semen samples. According to the pre-embryo grading standard proposed by Veeck et al. [25], We rate the development of embryo on the third day. The embryo can be divided into 5 grades. First grade, blastomere is uniform, without debris; the second grade, the blastomere is evenly distributed with small fragments; the third grade, the blastomere is uneven, no fragment; the fourth grade, the blastomere is uniform or uneven with many fragments; the fifth grade, a lot of embryo fragments, almost no recognition of blastomeres. We counted the number of cells on the third day greater than or equal to 6 cells in embryo, and the embryos with a score of 3 or higher are rated as effective embryos(EE), and the number of the third embryo cells is greater than or equal to 7 cells and the embryos with a score greater than or equal to 2 are evaluated as high quality embryos(HQE) .

### RNA library preparation and sequencing

We extracted total cellular RNA using TRIzol reagent (Takara) (with 40 μM DTT) and constructed a small RNA library using approximately 200 ng of total cellular RNA. High-throughput RNA sequencing was performed on a HiSeq 2000 system (SE50). Cutadapt was used to edit the adapter and to filter low-quality reads. Reads that did not match the adaptor or were less than 17 nt in length were discarded. To assess the expression levels of the miRNAs, only reads that exactly matched the 5′ initiation site of the annotated miRNA, those with ≤2 nt deletion at the 3′ terminus or those from the pri-miRNA were considered miRNAs. The reads of individual miRNAs were divided by the total reads aligned to the human genome and expressed as RPM for normalization. All data used to obtain the conclusions are presented in the paper. Sequencing data have been deposited in the National Center for Biotechnology Information Gene Expression Omnibus (GEO) (http://www.ncbi.nlm.nih.gov/geo/) under accession number GSE137182.

### Statistical analysis

All statistical analyses were performed using R statistical software. Regarding the ratios linked to the sequencing data, a density plot with rugs was generated to show their distributions. Heat maps were generated to visualize the expression values of all samples using the gplots package in R. Analysis of variance was used to identify the differentially expressed genes in various FR (fertilization rate), EER (effective embryo rate**)** and HQER (high-quality embryo rate) groups. The edgeR package in R was used to identify differentially expressed genes. Genes were considered statistically significant if their adjusted *p* value was less than 0.05. Moreover, a Venn diagram was generated to discover the common differentially expressed genes in three groups with the VennDiagram package in R. To determine the difference in the hsa-mir-191-5p expression value between different FR (as well as EER and HQER) subgroups, we performed a series of Student’s t tests and displayed them as boxplots. In addition, ROC curve analysis was utilized in this process to validate the results with the Daim package in R. To confirm this finding, we further performed a regression analysis of the hsa-mir-191-5p expression levels in the FR, EER and HQER groups, and the Pearson test was used to test the linear relationship.

## Supplementary information

**Additional file 1:** MicroRNAs expression matrix.

## Data Availability

The datasets generated and analysed during the current study are available in the GEO repository,ACCESSION NUMBER TO DATASETS:GSE137182. WEB LINK:https://www.ncbi.nlm.nih.gov/geo/query/acc.cgi?acc=GSE137182

## Abbreviations

DALRD3: DALR anticodon binding domain containing 3
EER: Effective embryos rate
FR: Fertilization rate
GEO: Gene Expression Omnibus
HQER: High quality embryos rate
hsa: Human sapiens
ICSI: Intracytoplasmic sperm injection
IVF: In vitro fertilization
miR or miRNA: MicroRNA
mmu: *Mus musculus*
NDUFAF3: NADH Dehydrogenase [Ubiquinone] 1 Alpha Subcomplex Assembly Factor 3
QC: Quality control
*ROC*: Receiver operating characteristic curve
RPM: Reads of exon model per million mapped reads
SCNT: Somatic cell nuclear transfer
WHO: World health organization
